# Supplementary material for: Stage-differentiated ensemble modeling of DNA methylation landscapes uncovers salient biomarkers and prognostic signatures in colorectal cancer progression
Source: PLoS One. 2022 Feb 24;17(2):e0249151. doi: 10.1371/journal.pone.0249151 (PMC8870460; doi:10.1371/journal.pone.0249151)
Supplement: Attachment — Submitted filename: response2reviewers.pdf [file pone.0249151.s002.pdf]

>>>At the outset, we would like to thank the reviewers and the Editor for their valuable comments.

The manuscript has been reviewed by two experts in the filed that both found it quite good and of interest, despite some problems, highlighted in particular by R.2, that must be addressed before it can be considered for publication.
this has been done.

>>>We have now substantially revised the manuscript and expanded the scope of our investigations / discussion.

1. Please ensure that your manuscript meets PLOS ONE's style requirements, including those for file naming.
>>> done

2. Please include captions for your Supporting Information files at the end of your manuscript, and update any in-text citations to match accordingly. Please see our Supporting Information guidelines for more information: http://journals.plos.org/plosone/s/supporting-information.
>>>This has been done.

Reviewer #1: In this paper Muthamilselvan et al., developed a comprehensive computational framework for stage-differentiated modelling of DNA methylation landscapes in CRC, found significant changes and discovery a novel CIMP-like signature
bearing potential clinical significance.
The data are supported by a strong statistical analysis.
The paper can be acceptted for pubblication.
Minor point
+Page 3 the word "Tomczak" must be deleted.

>>> Thank you. We would like to thank Reviewer #1 for their time and the kind comments.

Reviewer #2: In this study, the Authors propose a computational workflow for the analysis of the DNA methylation aberrations in colorectal cancer (CRC) with a stage-differentiated perspective. Data have been collected from The Cancer Genome Atlas (TCGA) portal; as a result, the Authors identify 7 stage-characteristic genes that could be indicative of a novel CpG island methylator phenotype (CIMP).
The manuscript provides an interesting perspective and a detailed explanation of how DNA methylation data could be analyzed to detect epigenetic signatures between normal and tumoral samples or in different stages of the disease. Bioinformatic procedures are well described and depicting a useful workflow when handling big and complex data from repositories such as TCGA.
Unfortunately, however, I cannot avoid pointing out that the work has serious shortcomings that do not allow to accept its publication in the present version and must be mandatorily corrected.
Importantly, even if interesting, data are presented in a confused manner; in particular, the Authors should better define the aim of the study and the experimental design, carefully organize the Results, improve the Discussion and avoid exaggerated conclusions, concerning in particular inferences drawn from data that should be better supported by experimental validation. Specific comments are listed below.

>>> We would like to thank Reviewer #2 for the careful reading of our paper and the critical comments. We have addressed all the many valid points in the present revision. We have undertaken a major revision of the manuscript in line with the suggestions.

1. The Authors should carefully revise the text and correct some grammar mistakes.
>>> yes
2. Figures are in many cases blurred and not legible; their quality must be improved.
>>> all figures have been redone in high-resolution tiff format
3. The Authors should pay attention to some typing mistakes and font differences (see, for example, pages 14 and 12).
>>> yes
4. TCGA collects data from at least 10 different studies on CRC; at least the cohort from which data have been collected should be reported in Materials and Methods.
>>> this has been identified and recorded in the manuscript under Methods.
5. TCGA collects data from 236 patients profiled with Human Methylation Bead Chip HM27, and 393 with HM450, measuring 27,000 and 480,000 CpG sites, respectively; why only the HM27 data have been used?
>>> This point has also been addressed in the Methods section. Essentially 450k Chips show enrichment in gene body and intergenic regions. A distribution of the CpG sites with respect to the genomic / genic location clearly indicates this (data not shown). 27k data are enriched in CpG sites in promoter regions. Please refer the section under Methods.
6. The correlation analyses between methylation and gene expression data are providing interesting information but should be better described by focusing the attention not only on the methodological procedure used but also on the biological meaning of the observed results.
>>> This has now been rectified. Indeed, we now show plots for only the stage-salient genes, to make the biological connections and meaning clear. We note that all the results from our investigations are available in the Supplementary Files.
7. In the Conclusions the Authors write: “All the stage-salient genes were found to be hypermethylated, indicating a novel CIMP-like character possibly promoting epigenetic destabilisation, which in turn would drive the progression of colorectal cancer”. First, it is not clear where the hypermethylation associated with these stage-salient genes is located (promoter, TSS, CpG island or gene body); this should be better explained. Then, the role of the stage-salient genes identified by the Authors should be better characterized in the context of CRC to indicate a possible novel CIMP-like phenotype; I would suggest the Authors to enrich the Discussion by adding more details and experimental evidence of the involvement of these genes in CRC pathogenesis.
>>> We have now increased the literature weight for these statements and discussion. We have included a new Table 8 with the location of the Dm probes. and a new Figure 19 to support all these assertion. We also found a new publication citing stage-IV specificity for FAM123A while this manuscript was under review (medrxiv preprint of our work was available in October 2020).
Thank you.
