# Supplementary material for: Stage-differentiated ensemble modeling of DNA methylation landscapes uncovers salient biomarkers and prognostic signatures in colorectal cancer progression
Source: PLoS One. 2022 Feb 24;17(2):e0249151. doi: 10.1371/journal.pone.0249151 (PMC8870460; doi:10.1371/journal.pone.0249151)
Supplement: Attachment — Submitted filename: Response to Reviewers_R4.pdf [file pone.0249151.s005.pdf]

Response to Reviewers:

>>> We would like to thank the Academic Editor and the Reviewers for helping improve our manuscript. All figures have individually passed the PACE digital diagnostic tool, https://pacev2.apexcovantage.com/ to ensure meeting PLOS requirements. References have been thoroughly checked once again.

Reviewer #3: This is an interesting and potentially useful study where a comprehensive computational framework for modelling of the stage-associated DNA methylation in colorectal cancer (CRC) was developed. The authors have addressed previous critiques.

>>> Thanks.

Reviewer #4: While the authors have addressed few of the comments from the previous revisions, a few minor points mainly about the cohort remain to be addressed in a greater detail-

1. As pointed out previously, there are various TCGA CRC the authors however do not clearly mention the details of the cohort.

2. It is briefly mentioned how the samples were from studies from Johns Hopkins or UCSC studies but it is unclear if these are two different studies from these centers (hence raising a question of batch effect) or if this was a combined effort. The authors should consider adding more information about the cohort.

>>> The comments are related, and we would like to thank the reviewer for raising this. The 27k methylation data were all shipped and processed by a **single organization** with code:05 JHU_USC center (Johns Hopkins – Univ. Southern California). This ensures homogeneity in data processing and submission to the TCGA. But the tissue source sites could be numerous (see below), so we carried out an analysis of batch-effects (https://bioinformatics.mdanderson.org/public-software/tcga-batch-effects/).

This yielded a Dispersion Separability Criterion of 0.299, which is < 0.5 and much lower than the recommended threshold of 1.0 for batch-correction. At the minimum, DSC values need to be >0.5 to consider the possibility of batch effects existing in the data. Thus, the samples within batches are as homogeneous to each other only as the batches themselves are to each other.

For instance, there were 29 tissue source sites for COAD alone:

1. Albert Einstein Medical Center
2. Mary Bird Perkins Cancer Center - Our Lady of the Lake
3. Duke University
4. University of Sao Paulo
5. Christiana Healthcare
6. Indivumed
7. International Genomics Consortium
8. Cureline
9. St. Joseph's Medical Center-(MD)
10. UNC
11. University of Pittsburgh

12. ILSBio
13. Harvard
14. MSKCC
15. Greater Poland Cancer Center
16. University Of Michigan
17. Asterand
18. Roswell Park
19. Candler
20. BLN - Baylor
21. University of Chicago
22. CHI-Penrose Colorado
23. Northwestern University
24. St. Joseph's Hospital AZ
25. Medical College of Georgia
26. Molecular Response
27. Institute of Human Virology Nigeria
28. University of Kansas
29. Wake Forest University

>>>We would like to thank the AE and Reviewers again for their comments.
