# Supplementary material for: Stage-differentiated ensemble modeling of DNA methylation landscapes uncovers salient biomarkers and prognostic signatures in colorectal cancer progression
Source: PLoS One. 2022 Feb 24;17(2):e0249151. doi: 10.1371/journal.pone.0249151 (PMC8870460; doi:10.1371/journal.pone.0249151)
Supplement: Attachment — Submitted filename: Response to Reviewers_R2.docx [file pone.0249151.s003.docx]

Response to Reviewers:

We would like to thank the Editor and Reviewers for their comments. We have updated all the figures again, to ensure maximum clarity, and also subjected each individual figure to the Preflight Analysis and Conversion Engine (PACE) digital diagnostic tool, <https://pacev2.apexcovantage.com/> to ensure that every individual figure meets PLOS requirements. We can assure you that all figures meet the requirements. If there is anything wanting in any figure, please let us know the figure identification and we will rectify it immediately.

As there are no other comments in the Decision Letter, we are re-submitting the manuscript for publication. Thank you.
